# *FGD1*-related Aarskog–Scott syndrome: Identification of four novel variations and a literature review of clinical and molecular aspects

**DOI:** 10.1007/s00431-024-05484-9

**Published:** 2024-02-27

**Authors:** Sujuan Li, Anran Tian, Yu Wen, Wei Gu, Wei Li, Xiaohong Qiao, Cai Zhang, Xiaoping Luo

**Affiliations:** 1 Department of Pediatrics, Tongji Hospital, Tongji Medical College, Huazhong University of Science and Technology, Wuhan, 430030 People’s Republic of China; 2 Department of Pediatrics, The First People’s Hospital of Urumqi, Urumqi, 830000 People’s Republic of China; 3 Department of Endocrinology, Children’s Hospital of Nanjing Medical University, Nanjing, 210008 People’s Republic of China; 4 Department of Pediatrics, Tongji Hospital, Tongji University School of Medicine, Shanghai, 200065 People’s Republic of China

**Keywords:** Aarskog–Scott syndrome, *FGD1*, Short stature, Growth hormone treatment, Genotype–phenotype correlation analysis

## Abstract

Patients with Aarskog–Scott syndrome (AAS) have short stature, facial anomalies, skeletal deformities, and genitourinary malformations. FYVE, RhoGEF, and PH domain-containing 1 (*FGD1*) is the only known causative gene of AAS. However, the diagnosis of AAS remains difficult, and specific treatments are still absent. Patients suspected with AAS were recruited, and clinical information was collected. Genetic testing and functional analysis were carried out for the diagnosis. By literature review, we summarized the clinical and genetic characteristics of *FGD1*-related AAS and analyzed the genotype–phenotype correlation. Five patients were recruited, and four novel *FGD1* variants were identified. The diagnosis of AAS was confirmed by genetic analysis and functional study. Three patients treated with growth hormone showed improved heights during the follow-up period. By literature review, clinical features of AAS patients with *FGD1* variants were summarized. Regarding *FGD1* variations, substitutions were the most common form, and among them, missense variants were the most frequent. Moreover, we found patients with drastic variants showed higher incidences of foot and genitourinary malformations. Missense variants in DH domain were related to a lower incidence of cryptorchidism.

*Conclusion*: We reported four novel pathogenic *FGD1* variations in AAS patients and confirmed the efficacy and safety of growth hormone treatment in *FGD1*-related AAS patients with growth hormone deficiency. Additionally, our literature review suggested the crucial role of DH domain in FGD1 function.

**Table:** 

**What is Known:** *• Aarskog–Scott syndrome is a rare genetic disease, and the only known cause is the variant in FGD1 gene. The typical clinical manifestations of AAS include facial, skeletal, and urogenital deformities and short stature.*	
**What is New:** *• We reported four novel FGD1 variants and reported the treatment of growth hormone in FGD1-related AAS patients. Our genotype–phenotype correlation analysis suggested the crucial role of DH domain in FGD1 function.*

**Supplementary Information:**

The online version contains supplementary material available at 10.1007/s00431-024-05484-9.

## Introduction

Aarskog–Scott syndrome (AAS, OMIM #305400), or faciogenital dysplasia (FGDY), firstly described by Aarskog in 1970 and further improved by Scott in 1971, is a rare genetic disease with a population prevalence of about 1/25,000 [1–3]. The clinical manifestations of AAS are heterogeneous. Typical features include short stature, facial anomalies (hypertelorism, ptosis, anteverted nares, and long philtrum), skeletal deformities (short, broad hands, and short fifth fingers), and genitourinary malformations (shawl scrotum, cryptorchidism, and inguinal/umbilical hernia) [4]. Rare symptoms such as cardiovascular defects, mild mental retardation, and attention-deficit hyperactivity disorder (ADHD) have also been reported [5–8].

Although there were a few reports of different inheritance patterns in AAS, the majority cases are inherited in X-linked recessive pattern. *FGD1* (FYVE, RhoGEF, and PH domain-containing 1) gene located on chromosome Xp11.21 is responsible for part of the X-linked recessive form of AAS and is also the only known causative gene of AAS [3]. *FGD1* consists of 18 exons, encoding FGD1 protein of 961 amino acids. The FGD1 protein contains five important domains including a putative Src homology 3 (SH3)-binding region (PRD), two pleckstrin homology (PH) domains, a Dbl homology (DH) domain, and a zinc finger (FYVE) domain [9]. It is a Rho/RAC guanine nucleotide exchange factor (GEF) and can regulate many physiological functions, such as cytoskeleton remodeling, osteoblast differentiation, extracellular matrix remolding, and cell trafficking [5, 10–12]. Importantly, studies have reported that FGD1 played a critical role in skeletal development via regulating downstream factors including extracellular signal-regulated kinase 1/2 (ERK1/2) and runt-related transcription factor 2 (RUNX2) in osteoblasts [11].

Although *FGD1* variant is the only known cause of AAS, there are only about 20% of AAS patients who were confirmed to have variants in *FGD1*, which may be due to the presence of undetected *FGD1* variants or the involvement of epigenetic or exogenous causes in the pathogenesis of AAS [5, 13]. Because it is easily confused with other syndromes like Noonan syndrome and Robinow syndrome, the diagnosis is very difficult without clear biochemical diagnostic criteria. In addition, as the patients grow older, the phenotype may become less typical, making diagnosis more difficult [7]. Moreover, due to the unclear pathogenesis of AAS, there is a lack of specific treatment for the disease, and the therapeutic effect is also unclear currently.

In this study, we recruited five Chinese patients with suspected AAS and confirmed the diagnosis by next-generation sequencing and functional study. The follow-up data of recombinant human growth hormone (rhGH) treatment on *FGD1*-related AAS patients were presented. Moreover, combined with the literature review of AAS patients with *FGD1* variants, the correlations between genotype and phenotype were analyzed.

## Materials and methods

### Patient enrollment and clinical investigations

Patients who presented with suspected AAS and showed clinical manifestations including short stature, facial anomalies, skeletal deformities, and genitourinary malformations were enrolled in this study. The medical history and clinical information of patients were collected. The serum IGF-1 and IGFBP-3 levels were detected by chemiluminescent immunoassay (IMMULITE 2000 XPi immunoassay system, Siemens Ltd), and the IGF-1 SDS was calculated as previously reported [14]. A combined insulin–clonidine or arginine–clonidine stimulation test was conducted in the patients to assess the peak level of growth hormone secretion. The stimulated growth hormone level greater than 10 ng/mL is considered to be normal [15]. During the treatment of rhGH, the patients were followed up every 3 to 4 months. At each visit, the height and weight were monitored. The serum IGF-1 levels and fasting blood glucose and insulin levels, as well as thyroid function, were evaluated at the same time.

### Ethics statement

The human study was approved by the Ethics Committee of Tongji Hospital, Tongji Medical College, Huazhong University of Science and Technology (TJ-IRB20180703). The study was performed according to the Declaration of Helsinki. Written informed consents were obtained from the parents.

### Genetic testing

Peripheral blood was collected from the patients and their family members. The genomic DNA was extracted from each blood sample by QIAamp DNA Blood Mini Kit (Qiagen GmbH, Hilden, Germany). To perform next-generation sequencing, the genomic DNA was fragmented, and the DNA library was prepared by MyGenostics protocols (Beijing, China). The amplified DNA was captured using the GenCap WES capture kit (MyGenostics, Beijing, China). The enrichment libraries were then sequenced on the DNBSEQ-T7 platform (MGI, Shenzhen, China). After sequencing, the raw data was analyzed by the BWA-GATK procedure (http://bio-bwa.sourceforge.net/ and https://software.broadinstitute.org/gatk/). Variants were further annotated by ANNOVAR (http://annovar.openbioinformatics.org/en/latest/) and associated with multiple databases: the 1000 Genomes Project (http://www.1000genomes.org), bSNP (http://www.ncbi.nlm.nih.gov/projects/SNP), the Human Gene Mutation Database (HGMD) (http://www.hgmd.cf.ac.uk), and the MyGenostics local database.

To confirm the *FGD1* variants from next-generation sequencing, corresponding exons and exon–intron boundaries were amplified by PCR and purified and then sequenced using the ABI3730XL sequencer (Applied Biosystems; Thermo Fisher Scientific, Inc., Waltham, MA, USA). The sequence data was then analyzed using Mutation Surveyor DNA Variant Analysis software (version 4.0.4; SoftGenetics, LLC.).

### Bioinformatic analysis of the function of identified variants

SIFT (http://provean.jcvi.org/index.php), PolyPhen-2 (http://genetics.bwh.harvard.edu/pph2), MutationTaster (http://www.mutationtaster.org), Combined Annotation Dependent Depletion (CADD) Phred scores (https://cadd.gs.washington.edu/), and GERP++ (https://mendel.stanford.edu/sidowlab/downloads/gerp/index.html) were used to predict the effect of identified variants on protein function. Protein multi-sequences alignment and amino acid hydrophilicity analysis were displayed by DNAMAN 6.0.3.99 (Lynnon Biosoft, USA).

### Plasmid construction

Wild-type human *FGD1* cDNA was subcloned into the eukaryotic expression vector pcDNA3.1-Flag-C as the template for further cloning steps. Based on the wild-type plasmid, site-directed mutagenesis was performed using the QuikChange site-directed mutagenesis kit (Stratagene, La Jolla, CA, USA) to construct the mutant plasmids. Each of the plasmids was verified by DNA sequencing. The sequencing primers used are listed in Table S1.

### Cell culture and transfection

HEK-293T cells were cultured in DMEM supplemented with 10% fetal bovine serum and 1% penicillin-streptomycin at 37 ℃ in a humidified 5% CO_2_ incubator. Cells were seeded in 12-well plates, and when the cellular fusion rate was 70–80%, 2 µg plasmid DNA and 4 µl Lipo3000 Reagent (Invitrogen, USA) were added to each well according to the manufacturer’s instruction. Meanwhile, pCMV6-AC-GFP was co-transfected with *FGD1* vectors as a control of transfection. After 48 h of transfection, HEK-293T cells were collected and further analyzed.

### Quantitative real-time PCR

Total RNA was extracted from transfected cells using RNAiso plus (Takara, Tokyo, Japan), and RNA was reverse-transcribed using PrimeScript RT Master Mix (Takara, Tokyo, Japan) according to the manufacturer’s protocol. All amplifications were performed on CFX Connect Real-Time PCR Detection System (Bio-Rad, Hercules, CA, USA) with a 10 µl reaction mixture consisting of 0.2 µl forward primer, 0.2 µl reverse primer, 1 µl cDNA, 3.6 µl H_2_O, and 5 µl SYBR Premix EX Taq (Takara, Tokyo, Japan). *GFP* was amplified as an internal control. The comparative threshold cycle (2^−ΔΔCT^) method was used to analyze the relative mRNA expression. The primers for RT-qPCR are provided in Table S1.

### Western blot

Protein extracted from the transfected cells with RIPA lysis buffer (BOSTER, Wuhan, China) was separated by 10% SDS-PAGE and then transferred to nitrocellulose filter membranes. After blocking with 5% skim milk for 2 h at room temperature, the membranes were incubated overnight at 4 ℃ with primary antibodies as shown in Table S2. After incubating with the secondary antibodies, protein bands were visualized by a chemiluminescence system (ChemiDox XRS+, Bio-Rad, CA, USA) and then analyzed using Image Lab software from Bio-Rad.

### Literature search strategy and data analysis

Case series studies or case reports containing the clinical data and information of *FGD1* gene variations of AAS patients were searched in HGMD, PubMed, and two Chinese public searching databases (China National Knowledge Infrastructure and WANFANG DATA). The following search terms were used: Aarskog–Scott syndrome, *FGD1*, faciogenital dysplasia, faciodigitogenital syndrome, or Aarskog syndrome. The searching deadline was January 2024. Cases without genetic evidence were excluded. Cases without complete phenotypic descriptions were excluded when analyzing the phenotypic characteristics of AAS patients. Patient basic information, clinical characteristics, and *FGD1* gene variations were extracted from selected cases. Ultimately, the phenotypic spectrum and variant spectrum of *FGD1*-related AAS patients were obtained.

### Statistical analysis

The continuous variable was expressed as mean ± SEM, and the categorical variable was expressed as number and percentage. One-way analysis of variance (ANOVA) with Tukey’s multiple comparisons test was used for multiple comparisons of normally distributed data. Fisher’s exact test was used to compare the incidence of clinical characteristics between the two groups.* P* < 0.05 was considered to be statistically significant. When conducting pairwise comparisons between multiple groups, Bonferroni correction was used to correct for the significance levels. All statistical tests were performed with GraphPad Prism software version 8.0 (GraphPad Software Inc., San Diego, CA, USA).

## Results

### Case series

#### Clinical characteristics

This study enrolled five Chinese Han patients from four different medical centers in China. Except that case 2 and case 3 were brothers, the patients were not related. All of them were born to non-consanguineous parents. Cases 1–3 had family history of short stature, especially their mothers. No facial or skeletal malformations were observed in their parents.

Their basic clinical characteristics and laboratory examination at the first visit are shown in Table S3. They were all male aged from 4 to 10 years old. Other than case 4, the patients showed different patterns of intrauterine growth restriction in aspect of birth weight or birth length. Severe postnatal growth retardation was found in each of the patients. Except for case 2, delayed bone age was found in these patients. The IGF-1 and IGFBP-3 levels of the patients were normal. The stimulated growth hormone levels were normal except for case 5. All of the patients had normal blood cell counts, liver function, kidney function, serum electrolytes, and blood gas. Their fasting blood glucose and insulin, thyroid function, and adrenal function were also normal.

**Fig. 1:**
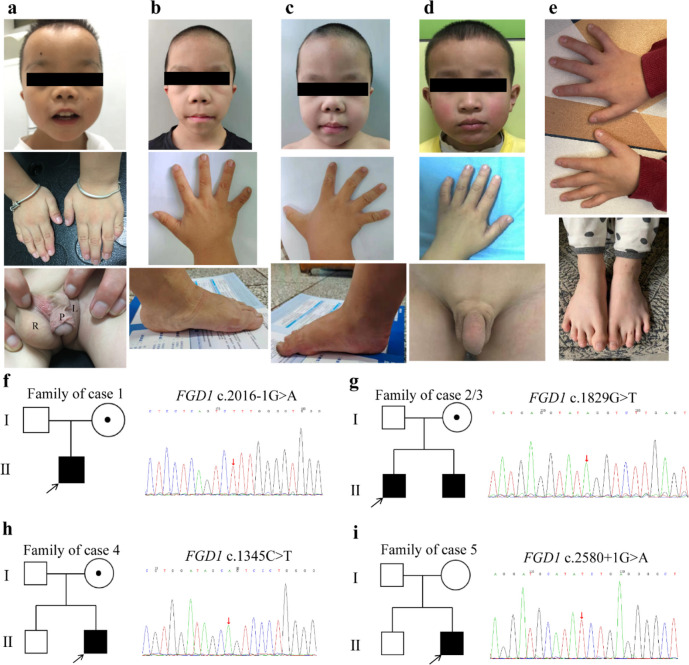
Phenotypes, pedigree, and gene sequencing results of the patients we recruited. **a** The facial features (frontal bossing, short nose with anteverted nares and long philtrum, a lower ear position), short fingers, curved fifth fingers, and urogenital abnormalities of case 1. P, the penile; L, the left scrotum; R, the right scrotum. **b**, **c** The facial features (frontal bossing, short nose with anteverted nares and long philtrum), short fingers (especially the fifth fingers), and flatfoot of case 2 and case 3. **d** The facial features (frontal bossing, widow’s peak, and small jaw), short fingers (especially the fifth fingers), bilateral cryptorchidism of case 4. **e** The short fingers and bulbous toes of case 5.** f–i** Pedigree of 4 families in our study and sequencing results of the *FGD1* variants. Squares indicate male family members; circles, female members; open symbols, unaffected members; black symbols, affected members with AAS characteristics; circles with a central dot, obligatory carriers; black arrow, proband

All of the patients showed typical features of AAS, such as short stature, abnormal facial features, skeletal deformities, and genitourinary malformations. The AAS-related characteristics were summarized in Table 1, and some clinical characteristics were demonstrated in Fig. 1a–e.

**Table 1:** Genetic and clinical information of five patients in our study

	**Case 1**	**Case 2**	**Case 3**	**Case 4**	**Case 5**
**Genetic features**					
Variant effect	Splicing	Missense	Missense	Missense	Splicing
Nucleotide change	c.2016-1G > A	c.1829G > T	c.1829G > T	c.1345C > T	c.2580+1G > A
Amino acid change	/	R610L	R610L	R449C	/
Variant site	Intron 12	Exon 10	Exon 10	Exon 7	Intron 17
Affected domain	PH1	PH1	PH1	DH	PH2
Source of variant	Mother	Mother	Mother	Mother	De novo
**Growth retardation**					
Short stature	+	+	+	+	+
Delayed bone age	+	+	+	+	+
**Specific facial features**					
Hypertelorism	+	+	+	+	-
Short nose with anteverted nares/long philtrum	+	+	+	+	+
Ptosis	+	-	-	+	+
Downward slant of palpebral fissures	-	-	-	+	-
Widow’s peak	-	-	-	+	-
Frontal bossing	+	+	+	-	-
Dental malocclusion	-	+	+	-	+
**Skeletal deformities**					
Short, broad hands	-	+	+	+	+
Short fifth fingers/clinodactyly	+	+	+	+	+
Broad feet with bulbous toes	-	-	-	-	+
Flatfoot	-	+	+	-	+
Pectus excavatum	-	-	-	-	-
**Genitourinary malformations**					
Cryptorchidism	+	+	-	-	+
Inguinal hernia	+	-	-	-	-
Shawl scrotum	+	-	-	+	-
Hypospadia	+	-	-	-	-
**Neuropsychiatric disorders**	-	Mild intellectual disabilities	-	-	Attention disorder
**Cardiovascular defects**	PDA	-	PFO	-	ASD
**Follow-up data**					
Formulation of rhGH	/	Daily rhGH	Daily rhGH	/	PEG-rhGH
Therapeutic dose	/	0.05 mg/kg/day	0.05 mg/kg/day	/	0.2 mg/kg/week
Follow-up time	/	16 months	16 months	/	34 months
Height before treatment	/	118.5 cm (< 3rd, − 3.75 SDS)	101.6 cm (< 3rd, − 2.91 SDS)	/	119.3 cm (< 3rd, − 2.14 SDS)
Annual growth velocity before treatment	/	/	/	/	5 cm/year
Height after treatment	/	127.5 cm (< 3rd, − 2.99 SDS)	110.3 cm (< 3rd, − 2.14 SDS)	/	145.2 cm (25–50th, − 0.46SDS)
Annual growth velocity after treatment	/	6.75 cm/year	8.70 cm/year	/	8.68 cm/year

+ characteristic present, - characteristic absent, *PDA* patent ductus arteriosus, *PFO* patent foramen ovale, *ASD* atrial septal defect

Among them, case 1 had the most severe genitourinary malformations. He showed incomplete penoscrotal transposition and shawl scrotum. His penis was poorly developed and bent downward, with the absence of a penile frenulum. He also had hypospadias with the urethra opening on the ventral side of the penis close to the scrotum. Moreover, a bifid and empty scrotum was found in him. He had cryptorchidism, and the bilateral testicles were palpable in the inguinal canals. An inguinal hernia was also found in him with a reversible mass in the right scrotum. In addition, case 2 was the elder brother of case 3, and both of them had similar facial features. Differently, case 2 had cryptorchidism on the right side, mild defect in intelligence, and normal cardiac ultrasonography, while case 3 showed no genitourinary abnormalities, normal intelligence, and patent foramen ovale on the ultrasonography. Case 4 had no other symptoms except for typical AAS phenotypes such as short stature, short nose with anteverted nares/long philtrum, ptosis, short fingers, and shawl scrotum. As for case 5, in addition to typical AAS symptoms, he also had attention disorder and atrial septal defect, which were rare clinical phenotypes of AAS.

#### Genetic testing and bioinformatic analysis

In these patients, four novel variants in *FGD1* gene were found by next-generation sequencing and confirmed by Sanger sequencing in the patients and their parents (Figs. 1f–i, and S1; Table 1).

A maternal inherited splice-site variant of c.2016-1G>A and a *de novo* splice-site variant of c.2580+1G>A were found in case 1 and case 5, respectively. These two canonical splice-site variants were not reported in the population databases mentioned above. According to the guidelines of the American College of Medical Genetics (ACMG), both of the splice-site variants in *FGD1* were determined as pathogenic (PVS1+PM2+PP4 for case 1 and PVS1+PS2+PM2+PP4 for case 5).

**Fig. 2:**
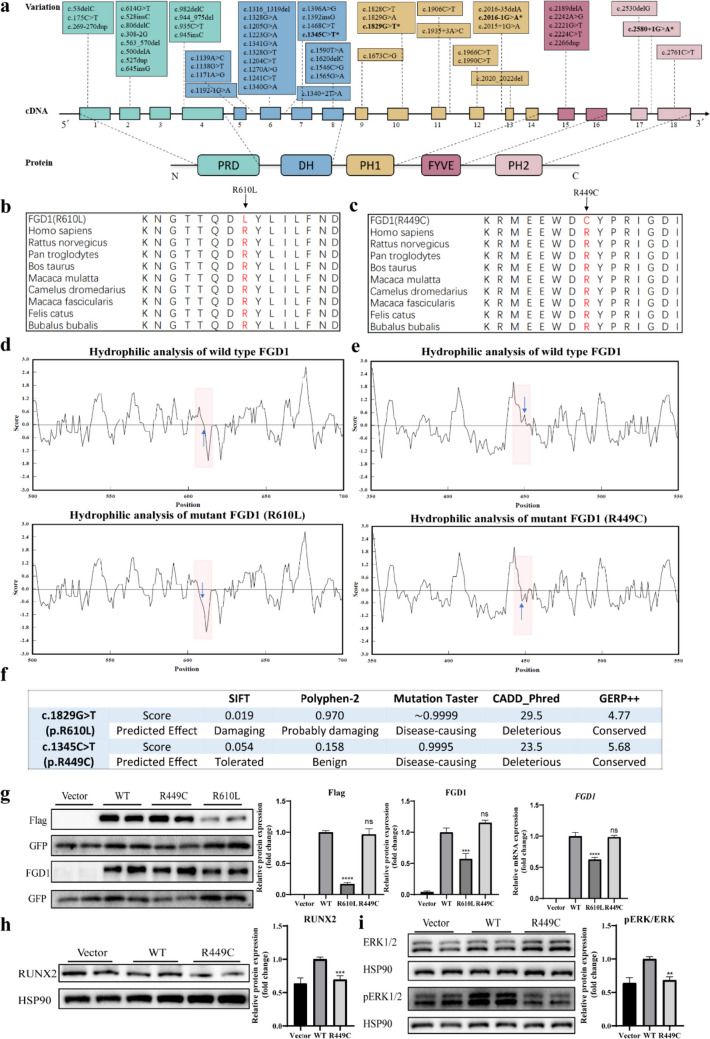
Functional analysis of two missense variants. **a** Schematic diagram of *FGD1* cDNA and protein with locations of identified variants. *, variants identified in this study. **b**, **c** Alignment of multiple FGD1 protein sequences across different species. The affected sites R610 and R449 are located in the highly conserved amino acid region. Red letters show the R610 and R449 sites. **d**, **e** The hydrophilicity analysis of wild-type and variant FGD1 proteins. Blue arrows show the R610 and R449 sites.** f** Interpretation of the novel missense variants by in silico predictive software.** g** The representative image of western blot and quantification analysis of Flag-FGD1, FGD1 protein, and relative *FGD1* mRNA expression in HEK-293T cells after transfected with empty vector (vector), wild-type plasmid (WT), or mutated plasmid (R610L and R449C). GFP co-transfected was used as a transfection control. **h**, **i** Western blot analysis of RUNX2 and pERK/ERK in HEK-293T cells. HSP90 was used as a control for protein loading. Data were expressed as mean ± SEM (*n* = 6), **P < 0.01, ***P < 0.001, ****P < 0.0001, *ns* means no significant difference

Maternal inherited missense variants of c.1829G>T (p.R610L) and c.1345C>T (p.R449C) were found in case 2/3 and case 4, respectively, and both of the variants were absent from population databases (PM2). Additionally, a missense variant at the same amino acid residue with c.1829G>T (p.R610L) but with a different amino acid change was reported to be pathogenic (PM5) [16]. The amino acids at both variant sites were highly conserved across different species (Fig. 2b, c). The hydrophilicity analysis showed reduced hydrophilicity of both mutated proteins compared to wild-type FGD1, suggesting potential changes in the structure or function of the protein (Fig. 2d, e). Moreover, multiple in silico tools indicated the deleterious effects of both missense variants on the gene products (Fig. 2f). According to the ACMG guidelines, the missense variant of c.1829G>T (p.R610L) was determined as likely pathogenic (PM2+PM5+PP3+PP4), while the missense variant of c.1345C>T (p.R449C) was determined as uncertain (PM2+PP4).

#### In vitro functional analysis of the identified missense variant*s*

To further investigate the pathogenicity of the identified missense variants, mutated *FGD1* vectors of R610L and R449C were constructed. Wild-type (WT) or mutated *FGD1* vectors were transfected into HEK-293T cells together with pCMV6-AC-GFP as an external control of transfection.

Firstly, the effect of R610L mutant on FGD1 mRNA and protein expression was analyzed. In cells transfected with R610L mutant, the mRNA and protein levels of FGD1 were significantly suppressed by R610L mutant compared with WT vectors (Fig. 2g). It suggested that R610L mutant could affect the stability of mRNA or the processing and modification process of mRNA precursors. The pathogenicity of c.1829G>T (p.R610L) in *FGD1* was determined as pathogenic (PS3+PM2+PM5+PP3+PP4).

In cells transfected with R449C mutant, both mRNA and protein expression levels of FGD1 remained similar with those in cells transfected with WT vectors (Fig. 2g). Thus, we further evaluated the expression levels of downstream effectors ERK 1/2 and RUNX2.

It revealed that the phosphorylation level of ERK 1/2 and protein expression level of RUNX2 were both significantly decreased in cells transfected with R449C mutant compared to cells transfected with WT vectors (Fig. 2h, i). Although the FGD1 protein expression was not affected, these results suggested that R449C mutant could disturb the cellular function of FGD1 indicated by RUNX2 and ERK 1/2. Thus, the pathogenicity of c.1345C>T (p.R449C) in *FGD1* was interpreted as likely pathogenic (PS3+PM2+PP4).

#### Treatment and follow-up

**Fig. 3:**
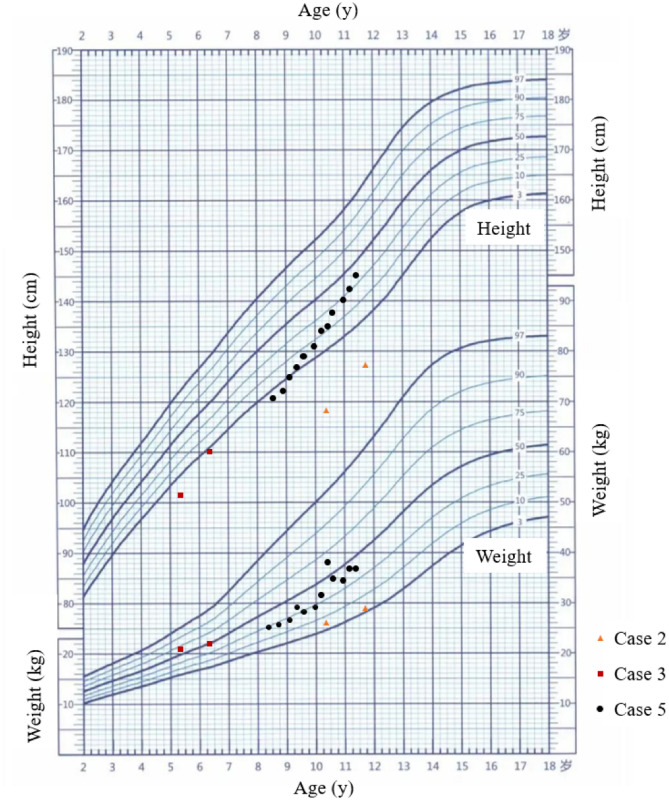
The growth curve of three patients during rhGH treatment

Based on the clinical manifestations and the results of genetic sequencing and functional analysis, the diagnosis of AAS was confirmed in these patients. With the consensus from the parents, case 2, case 3, and case 5 received rhGH treatment to improve their height. Among them, case 2 and case 3 were treated with daily rhGH for 16 months and 1 year, respectively, while case 5 was treated with once-weekly long-acting PEGylated rhGH (PEG-rhGH) for 34 months. The growth curve of 3 patients during rhGH treatment is shown in Fig. 3, and the detailed follow-up data is shown in Table 1.

For case 2 and case 3, they started the treatment immediately after the diagnosis (at the age of 10 years and 4 months and 5 years and 4 months, respectively). Before the treatment, their heights were 118.5 cm (<3rd, −3.75 SDS) and 101.6 cm (<3rd, −2.91 SDS), respectively, but their growth velocities were not recorded. The serum IGF-1 levels for cases 2 and 3 before the treatment were 218 ng/mL (−0.24 SDS) and 192 ng/mL (1.02 SDS), respectively. Case 2 was treated with daily rhGH of 0.05 mg/kg/day for 16 months. After the treatment, his height increased to 127.5 cm (<3rd, −2.99 SDS), and his estimated annual growth velocity during the treatment was 6.75 cm/year. Case 3 was treated with daily rhGH of 0.05 mg/kg/day for 1 year, and his height was 110.3 cm (<3rd, −2.14 SDS). His estimated annual growth velocity during the treatment was 8.70 cm/year. The IGF-1 levels of case 2 and case 3 increased to 448 ng/mL (1.51 SDS) and 236 ng/mL (1.54 SDS) after the treatments.

Case 5 was treated with the once-weekly PEG-rhGH for 34 months from the age of 8 years and 7 months. Before treatment, his growth velocity was about 5 cm/year, his height was 119.3 cm (< 3rd, −2.14 SDS), and his serum IGF-1 level was 200 ng/mL (0.23 SDS). After 16 months of PEG-rhGH treatment (0.2 mg/kg/week), his height increased to 131.2 cm (3rd to 10th, −1.45 SDS), and his annual growth velocity during the treatment was 7.95 cm/year, and the IGF-1 level increased to 382 ng/mL (1.73 SDS). After continuing treatment for 18 months, when he was 11 years and 5 months old, his height reached 145.2 cm (25–50th, −0.46 SDS), and his serum IGF-1 level reached 414 ng/mL (1.49 SDS). Throughout the entire treatment period, his annual growth velocity was 8.68 cm/year.

During the treatment, their glucose metabolism and thyroid function were all normal, and no related adverse events were reported. These results suggested the possible efficacy of rhGH treatment on the height of *FGD1*-related AAS children during the follow-up period. Longer follow-up time is needed to demonstrate the effectiveness and safety of rhGH treatment on *FGD1*-related AAS patients.

### Literature review of *FGD1*-related AAS

Since the variant in *FGD1* is currently the only clear cause of AAS, we analyzed *FGD1*-related AAS cases to explore the genotype and phenotype correlation. Including the four novel variants reported in our study, there were a total of 72 pathogenic or likely pathogenic variants reported. Among them, 62 variants were included in HGMD, 4 variants were found in the PubMed database, and 2 variants were found in the *Chinese Journal of Endocrine Metabolism* and the *Chinese Journal of Medical Genetics*. Additionally, 67 of the 72 reports described the clinical features of the affected subjects in detail, so we focused on these reports when analyzing the phenotypes of AAS patients.

#### Clinical features of *FGD1*-related AAS

Clinical features of 67 patients with *FGD1* variants were summarized in Table 2. The main features of AAS were short stature, facial anomalies, skeletal deformities, and genitourinary malformations. In our review, 98.4% of the patients showed short stature. Only one 16-year-old boy from Ireland reported to progress through puberty and attain a normal height (175 cm) [13]. The incidence of facial anomalies was 98.4%, and hypertelorism and short nose were the most common facial features. Skeletal deformities were indicated in 96.8% of the patients, and hand deformities were the most common skeletal deformities among them. Genitourinary malformations were present in 94.9% of the patients, and shawl scrotum was the most frequent followed by cryptorchidism. Moreover, the overall incidence of neuropsychiatric disorders was 51.5%, including developmental retardation, mental impairment, attention disorder, and mental retardation. There were 9 of the 66 patients (13.6%) had cardiovascular defects, such as ventricular septal defect, patent ductus arteriosus, vascular malformation, atrial septal defect, and patent foramen ovale. These results suggested that except for the main features of AAS, neuropsychiatric disorders and cardiovascular defects were also worthy noted in the patients.

**Table 2:** Summary of clinical features of patients with reported *FGD1* variations

**Clinical features**	*n*/*N*	%
**Short stature**	62/63	98.4
**Specific facial features**	61/62	98.4
Hypertelorism	56/62	90.3
Short nose	55/61	90.2
Long philtrum	46/61	75.4
Anteverted nares	45/61	73.8
Ptosis	38/61	62.3
Abnormal auricles/low ear position	31/61	50.8
Downward slant of palpebral fissures	29/61	47.5
Widow’s peak	25/61	41.0
Crease below the lower lip	17/61	27.9
Micrognathia	7/61	11.5
Maxillary hypoplasia	4/61	6.6
**Skeletal deformities**	60/62	96.8
Deformity of hand	60/62	96.8
Deformity of foot	38/62	61.3
Joint hyperextension	21/62	33.9
Thoracic skeletal malformation	9/62	14.5
**Genitourinary malformations**	56/59	94.9
Shawl scrotum	46/62	74.2
Cryptorchidism	37/62	59.7
Inguinal hernia	18/59	30.5
**Neuropsychiatric disorders**	34/66	51.5
Developmental retardation	16/66	24.2
Mental impairment	9/66	13.6
Attention disorder	9/66	13.6
Intellectual disabilities	9/66	13.6
**Cardiovascular defects**	9/66	13.6
Ventricular septal defect	3/66	4.5
Patent ductus arteriosus	2/66	3.0
Vascular malformation	2/66	3.0
Atrial septal defect	1/66	1.5
Patent foramen ovale	1/66	1.5

Due to the different emphasis on phenotypic description in individual literature, the total number of cases corresponding to each system was different

*n* the number of cases with corresponding phenotype, *N* the totalnumber of cases with corresponding symptom descriptions

#### Variant spectrum of *FGD1* gene

We analyzed the frequency of different types of *FGD1* variants. Substitutions were the most common form of *FGD1* DNA variants (64.2%), followed by deletion (16.4%) and insertion (7.5%) (Table 3). Structural variants were rare in *FGD1*, with only four reports of duplications and four reports of large-scale deletions. In respect to the effect on protein translation, missense variants were the most frequent form of variants (32.8%), followed by frameshift variants (28.4%) (Table 3). Moreover, as shown in Fig. 2a and Table 3, DH domain was the hotspot region for substitutions and indel variants (42.4%), suggesting the critical role of DH domain in the function of FGD1 protein. In addition, the number of variants occurring in the PRD and PH1 domains was second only to the DH domain, while the FYVE and PH2 domains exhibited fewer variants.

**Table 3:** Classification of variants based on DNA variants, variant effect, and domain distribution

	*n*	%
**DNA variants**		
SNVs (substitutions)	43	64.2
Deletions	11	16.4
Insertions	5	7.5
Duplications	4	6.0
Large-scale deletions	4	6.0
Total	67	100
**Variant effect**		
Missense variants	22	32.8
Frameshift variants	19	28.4
Nonsense variants	13	19.4
Splice-site variants	8	11.9
Gross deletions	4	6.0
Inframe deletion	1	1.5
Total	67	100
**Domain location of variants**		
PRD	14	23.7
DH	25	42.4
PH1	13	22.0
FYVE	4	6.8
PH2	3	5.1
Total	59 ^a^	100

*SNVs* single-nucleotide variants

^a^Excluding the structural variants (*n* = 8)

#### Genotype–phenotype correlation analysis

To compare the phenotype of individuals with different *FGD1* variants, we divided the patients into two groups according to the impact of variants on protein translation. One group included patients with missense variants (*n* = 22), and the other were patients with drastic variants (*n* = 43) (Table 4). The drastic variants included nonsense variants, frameshift variants, splice-site variants, and large deletion, which resulted in significant loss of the FGD1 protein function. The incidences of most features in the two groups were comparable. However, compared with the patients with missense variants, the incidences of foot deformities, shawl scrotum, and cryptorchidism were significantly higher in patients with drastic variants. These data implied that FGD1 protein was not only important for skeletal development but also indispensable for genital development.

**Table 4:** Genotype–phenotype correlation analysis

	Missense variants (*N* = 22)	Drastic variants (*N* = 43)	*P-*value
**Growth retardation**			
Short stature	19/19 (100.0%)	42/43 (97.7%)	> 0.99
Height SDS	− 3.235 (0.39)^a^	− 2.816 (0.21)^b^	0.31
**Specific facial features**	18/19 (94.7%)	42/42 (100.0%)	0.31
Hypertelorism	16/19 (84.2%)	39/42 (92.9%)	0.36
Short nose	16/19 (84.2%)	38/41 (92.7%)	0.37
Long philtrum	12/19 (63.2%)	33/41 (80.5%)	0.22
Anteverted nares	12/19 (63.2%)	32/41 (78.0%)	0.35
Ptosis	11/19 (57.9%)	27/41 (65.9%)	0.58
Abnormal auricles/low ear position	9/19 (47.4%)	21/41 (51.2%)	> 0.99
Downward slant of palpebral fissures	8/19 (42.1%)	21/41 (51.2%)	0.41
Widow’s peak	8/19 (42.1%)	16/41 (39.0%)	> 0.99
Crease below the lower lip	4/19 (21.1%)	12/41 (29.3%)	0.75
Micrognathia	2/19 (10.5%)	5/41 (12.2%)	> 0.99
Maxillary hypoplasia	1/19 (5.3%)	3/41 (7.3%)	> 0.99
**Skeletal deformities**	18/20 (90.0%)	41/41 (100.0%)	0.10
Deformity of hand	18/20 (90.0%)	41/41 (100.0%)	0.10
Deformity of foot	8/20 (40.0%)	29/41 (70.7%)	0.03*
Joint hyperextension	7/20 (35.0%)	14/41 (34.1%)	> 0.99
Thoracic skeletal malformation	2/20 (10.0%)	7/41 (17.1%)	0.70
**Genitourinary malformations**	15/20 (75%)	40/41 (97.6%)	0.01*
Shawl scrotum	11/20 (55%)	34/41 (82.9%)	0.03*
Cryptorchidism	7/20 (35%)	30/41 (73.2%)	0.006*
Inguinal hernia	6/19 (31.6%)	12/39 (30.8%)	> 0.99
**Neuropsychiatric disorders**	14/22 (63.6%)	19/43 (43.8%)	0.19
Developmental retardation	8/22 (36.4%)	7/43 (16.3%)	0.12
Mental impairment	3/22 (13.6%)	6/43 (14.0%)	> 0.99
Attention disorder	3/22 (13.6%)	6/43 (14.0%)	> 0.99
Intellectual disabilities	4/22 (18.2%)	5/43 (11.6%)	> 0.99
**Cardiovascular defects**	2/22 (9.1%)	7/43 (12.5%)	0.71
Ventricular septal defect	1/22 (4.5%)	2/43 (4.7%)	> 0.99
Patent ductus arteriosus	0	2/43 (4.7%)	0.55
Vascular malformation	0	2/43 (4.7%)	0.55
Atrial septal defect	0	1/43 (2.3%)	> 0.99
Patent foramen ovale	1/22 (4.5%)	0	0.34

Drastic variants include frameshift variants, nonsense variants, splice-site variants, large deletion/insertion, in which one case was excluded due to lack of detailed phenotypic description. *SDS* standard deviation score, height SDS were presented as mean (SEM)

*P < 0.05

^a^*n* = 6

^b^*n* = 12

We further analyzed the phenotype of different missense variants to compare the function of different FGD1 domains (Table 5). The results showed that the incidences of the majority features were comparable between missense variants located in different domains, especially for short stature and facial features. In the aspect of genitourinary malformations, the missense variants in DH domain were related with a significantly lower incidence of cryptorchidism compared with variants outside DH domain. Additionally, the missense variants in PRD domain had a higher incidence of mental impairment compared with variants outside PRD domain. Noteworthy, there were only two reported variants in PRD domain, and more studies are needed for further investigation.

**Table 5:** Correlation analysis between the affected domains and phenotypes

	Location of missense variants											
Phenotypes	In PRD domain (A)	Outside PRD domain (B)	*P-*value A vs. B	In DH domain (C)	Outside DH domain (D)	*P-*value C vs. D	In PH1 domain (E)	Outside PH1 domain (F)	*P-*value E vs. F	In FYVE domain (G)	Outside FYVE domain (H)	*P-*value G vs. H
**Short stature**	2/2 (100%)	17/17 (100%)	> 0.99	13/13 (100%)	6/6 (100%)	> 0.99	3/3 (100%)	16/16 (100%)	> 0.99	1/1 (100%)	18/18 (100%)	> 0.99
**Specific facial features**	1/2 (50%)	17/17 (100%)	0.11	13/13 (100%)	5/6 (83.3%)	0.32	3/3 (100%)	15/16 (93.8%)	> 0.99	1/1 (100%)	17/18 (94.4%)	> 0.99
Hypertelorism	1/2 (50%)	15/17 (88.2%)	0.30	11/13 (84.6%)	5/6 (83.3%)	> 0.99	3/3 (100%)	13/16 (81.3%)	> 0.99	1/1 (100%)	15/18 (83.3%)	> 0.99
Ptosis	1/2 (50%)	10/17 (58.8%)	> 0.99	8/13 (61.5%)	3/6 (50%)	> 0.99	2/3 (66.7%)	9/16 (56.3%)	> 0.99	0/1(0)	11/18 (61.1%)	0.42
Abnormal auricles/low ear position	1/2 (50%)	8/17 (47.1%)	> 0.99	7/13 (53.8%)	2/6 (33.3%)	0.63	1/3 (33.3%)	8/16 (50%)	> 0.99	0/1(0)	9/18 (50%)	> 0.99
Downward slant of palpebral fissures	1/2 (50%)	7/17 (41.2%)	> 0.99	5/13 (38.5%)	3/6 (50%)	> 0.99	1/3 (33.3%)	7/16 (43.8%)	> 0.99	1/1 (100%)	7/18 (38.9%)	0.42
Anteverted nares	1/2 (50%)	11/17 (64.7%)	> 0.99	8/13 (61.5%)	4/6 (66.7%)	> 0.99	2/3 (66.7%)	10/16 (62.5%)	> 0.99	1/1 (100%)	11/18 (61.1%)	> 0.99
Short nose	1/2 (50%)	15/17 (88.2%)	0.30	11/13 (84.6%)	5/6 (83.3%)	> 0.99	3/3 (100%)	13/16 (81.3%)	> 0.99	1/1 (100%)	15/18 (83.3%)	> 0.99
Long philtrum	1/2 (50%)	11/17 (64.7%)	> 0.99	9/13 (69.2%)	3/6 (50%)	0.62	2/3 (66.7%)	10/16 (62.5%)	> 0.99	0/1(0)	12/18 (66.7%)	0.37
Widow’s peak	0/2 (0)	8/17 (47.1%)	0.50	6/13 (46.2%)	2/6 (33.3%)	> 0.99	1/3 (33.3%)	7/16 (43.8%)	> 0.99	1/1 (100%)	7/18 (38.9%)	0.42
Crease below the lower lip	0/2 (0)	4/17 (23.5%)	> 0.99	3/13 (23.1%)	1/6 (16.7%)	> 0.99	1/3 (33.3%)	3/16 (18.8%)	0.53	0/1(0)	4/18 (22.2%)	> 0.99
Micrognathia	0/2 (0)	2/17 (11.8%)	> 0.99	2/13 (15.4%)	0/6(0)	> 0.99	0/3(0)	2/16 (12.5%)	> 0.99	0/1(0)	2/18 (11.1%)	> 0.99
Maxillary hypoplasia	0/2 (0)	1/17 (5.9%)	> 0.99	1/13 (7.7%)	0/6(0)	> 0.99	0/3(0)	1/16 (6.3%)	> 0.99	0/1(0)	1/18 (5.6%)	> 0.99
**Skeletal deformities**	1/2 (50%)	17/18 (94.4%)	> 0.99	13/14 (92.9%)	5/6 (83.3%)	0.52	3/3 (100%)	15/17 (88.2%)	> 0.99	1/1 (100%)	17/19 (89.5%)	> 0.99
Deformity of hand	1/2 (50%)	17/18 (94.4%)	> 0.99	13/14 (92.9%)	5/6 (83.3%)	0.52	3/3 (100%)	15/17 (88.2%)	> 0.99	1/1 (100%)	17/19 (89.5%)	> 0.99
Deformity of foot	0/2 (0)	8/18 (44.4%)	0.49	5/14 (35.7%)	3/6 (50%)	0.64	3/3 (100%)	5/17 (29.4%)	0.05	0/1(0)	8/19 (42.1%)	> 0.99
Joint hyperextension	1/2 (50%)	6/18 (33.3%)	> 0.99	6/14 (42.9%)	1/6 (16.7%)	0.3544	0/3(0)	7/17 (41.2%)	0.5211	0/1(0)	7/19 (36.8%)	> 0.99
Thoracic skeletal malformation	0/2 (0)	2/18 (11.1%)	> 0.99	2/14 (14.3%)	0/6(0)	> 0.99	0/3(0)	2/17 (11.8%)	> 0.99	0/1(0)	2/19 (10.5%)	> 0.99
**Genitourinary malformations**	1/2 (50%)	14/16 (87.5%)	> 0.99	10/12 (83.3%)	5/6 (83.3%)	> 0.99	3/3 (100%)	12/15 (80%)	> 0.99	1/1 (100%)	14/17 (82.4%)	> 0.99
Cryptorchidism	1/2 (50%)	6/18 (33.3%)	> 0.99	2/14 (14.3%)	5/6 (83.3%)	0.007*	3/3 (100%)	4/17 (23.5%)	0.03	1/1 (100%)	6/19 (31.6%)	0.35
Shawl scrotum	0/2 (0)	11/18 (61.1%)	0.19	8/14 (57.1%)	3/6 (50%)	> 0.99	2/3 (66.7%)	9/17 (52.9%)	> 0.99	1/1 (100%)	10/19 (52.6%)	> 0.99
Inguinal hernia	0/2 (0)	6/17 (35.3%)	> 0.99	6/13 (46.2%)	0/6(0)	0.11	0/3(0)	6/16 (37.5%)	0.52	0/1(0)	6/18 (33.3%)	> 0.99
**Neuropsychiatric disorders**	2/2 (100%)	12/20 (60%)	0.52	9/15 (60%)	5/7 (71.4%)	> 0.99	2/3 (66.7%)	12/19 (63.2%)	> 0.99	1/2 (50%)	13/20 (65%)	> 0.99
Developmental retardation	2/2 (100%)	6/20 (30%)	0.12	5/15 (33.3%)	3/7 (42.9%)	> 0.99	1/3 (33.3%)	7/19 (36.8%)	> 0.99	0/2(0)	8/20 (40%)	0.52
Mental impairment	2/2 (100%)	1/20 (5%)	0.01*	1/15 (6.7%)	2/7 (28.6%)	0.23	0/3(0)	3/19 (15.8%)	> 0.99	0/2(0)	3/20 (15%)	> 0.99
Attention disorder	0/2 (0)	3/20 (15%)	> 0.99	3/15 (20%)	0/7(0)	0.52	0/3(0)	3/19 (15.8%)	> 0.99	0/2(0)	3/20 (15%)	> 0.99
Intellectual disabilities	0/2 (0)	4/20 (20%)	> 0.99	2/15 (13.3%)	2/7 (28.6%)	0.56	1/3 (33.3%)	3/19 (15.8%)	0.47	1/2 (50%)	3/20 (15%)	0.34
**Cardiovascular defects**	1/2 (50%)	1/20 (5%)	0.18	0/16(0)	2/6 (33.3%)	0.06	1/3 (33.3%)	1/19 (5.3%)	0.26	0/2(0)	2/20 (10%)	> 0.99

*P < 0.0125

## Discussion

In this study, we reported five Chinese children with typical clinical characteristics of AAS. Four novel variants in *FGD1* were found in them, including two pathogenic canonical splice-site variants and two missense variants. We confirmed the diagnosis of AAS by bioinformatic analysis and cellular experiments according to the ACMG guidelines. With the consensus of their parents, three of them were treated with rhGH, and the follow-up data during growth hormone treatment were presented. Furthermore, we summarized the clinical and genetic characteristics of reported *FGD1*-related AAS patients and analyzed the genotype and phenotype association, which further deepened our understanding of AAS and *FGD1* gene.

The typical clinical manifestations of AAS included developmental malformations of the face, bones, and genitalia. As demonstrated in Table 1 and Fig. 1a–e, all five patients in our study exhibited these typical AAS symptoms. We further analyzed the incidences of these symptoms by literature review, and the results showed the incidences of short stature, special facial features, skeletal deformities, and genitourinary abnormalities were as high as 94.9–98.4% in *FGD1*-related AAS patients. As previously reported, neuropsychiatric disorders and cardiovascular abnormalities were considered as rare symptoms of AAS [5]. However, in our literature review, we found that the incidence of neuropsychiatric disorders was relatively high (51.5%), and the incidence of cardiac malformations reached 13.6% in *FGD1*-related AAS patients. Consistently, among the five patients in our case series, case 2 and case 5 had mild intellectual disabilities and attention disorder, respectively, and case 1, case 3, and case 5 all had different types of cardiovascular malformations. These results suggested that the neuropsychiatric disorders and cardiovascular malformations were worth to be noted in *FGD1*-related AAS patients.

Noteworthy, it has been reported that the clinical manifestations of patients with *FGD1* variations are heterogeneous [13]. Similarly, our literature review showed that the clinical phenotypes of patients with the same variant could be similar or completely different (Table S4). In our case series, although case 2 and case 3 had the same variant (R610L), they had slightly different symptoms as shown in Table 1, such as cryptorchidism, neuropsychiatric disorders, and cardiovascular defects. It suggested that other factors, like other genes, polymorphisms, epigenetic influences, and exogenous influences, could also have important effects on the phenotypes of AAS patients with *FGD1* variants.

Nowadays, the treatment for patients with AAS mainly focuses on the improvement of symptoms, and there is a lack of targeted treatment strategies. Growth hormone was reported to be used to promote growth in AAS patients [17]. However, the available data were still limited. There were only three *FGD1*-related AAS patients reported to receive rhGH treatment, with two patients having improved height [18–20]. In our study, rhGH treatment showed height improvement in the patients, and there were no serious adverse reactions during the treatment. However, the only patient in whom a longer follow-up was available and which still showed a good reaction in growth, was the one with an abnormal GH study, and that possibly rhGH suppletion should be limited to such patients. These results suggested the effectiveness and safety of rhGH for height improvement in AAS patients within a limited follow-up period. Still, longer follow-up time and further investigations are needed to confirm the therapeutic effect of rhGH on more AAS patients.

At present, the vast majority of AAS cases are caused by variations in *FGD1* gene located on X chromosome. In our study, single-nucleotide variations (SNVs) were the most common type of *FGD1* variants by literature review, accounting for 64.2% of all variants. Consistently, all *FGD1* variants in the 5 patients we reported were also SNVs. As the catalytic region of FGD1 protein, DH domain and its adjacent PH1 domain play the most important role in the GEF activity of FGD1, while other domains mainly play regulatory roles [21]. Further analysis of the domains corresponding to the variant sites revealed that DH domain was the hotspot region of *FGD1* variations, which further suggested the importance of DH domain. In addition, in our study, variant R449C, which located in the DH domain, affected the function of FGD1 protein by decreasing the expression level of downstream molecules of FGD1. Another variant, R610L, in the PH1 domain, reduced the expression level of FGD1 protein and affected its subsequent function. These results reflected the importance of the DH domain and PH1 domain to FGD1 protein function.

There were a few in-depth studies about the genotype and phenotype correlation of AAS, and they stated that there was no clear genotype–phenotype correlation. In 2010, Orrico et al. presented the clinical and genetic data from 11 patients with *FGD1* variations from a cohort of 60 AAS individuals studied, reporting 9 novel *FGD1* variations. They did not find any evidence for phenotype–genotype correlations between the variant type and position and clinical features [5]. In 2017, Ge et al. analyzed the clinical manifestations of patients with AAS carrying *FGD1* variants in different exons and introns but did not find a clear genotype–phenotype correlation [22]. Recently, Zanetti Drumond et al. summarized and analyzed the manifestations of 58 AAS patients with *FGD1* variants and pointed out that reported phenotypes did not present a direct relation to the underlying genotypes [23]. Differently, our analysis revealed that individuals with drastic variants might have higher incidences of foot deformities, shawl scrotum, and cryptorchidism than individuals with missense variants. These results indicated the significance of FGD1 protein function in skeletal and genitourinary development. In addition, we found that the incidence of cryptorchidism was relatively lower in patients with missense variants in DH domain compared with those with missense variants outside DH domain. We speculated that the missense variants occurring in DH domain were relatively mild variants with less disruption of FGD1 protein function. The reason why we draw different conclusions from other studies may be due to our different research methods, or the small number of our study individuals, and this will not hold in larger groups. In general, our analysis confirmed the importance of DH domain for FGD1 protein and deepened our understanding of the structure and function of *FGD1* gene.

The limitation of this study was the relatively small number of cases due to the rarity of the disease. Furthermore, given the limited data on family studies, we are currently unable to completely rule out the impact of ascertainment bias on the research results. More cases are needed for further detailed studies to explore the potential pathogenic mechanism, which would be helpful to find effective interventions for these patients. Furthermore, comparing the phenotypic differences among different ethnic groups would also be meaningful for diagnosing AAS patients.

## Conclusion

In this study, we reported four novel pathogenic *FGD1* variants in AAS patients and showed the potential efficacy and safety of rhGH treatment in improving height outcome of AAS patients. By literature review, we sorted out the phenotypic spectrum and variant spectrum of *FGD1*-related AAS patients. Further genotype–phenotype correlation analysis suggested the importance of FGD1 protein for skeletal and genitourinary development, as well as the significance of DH domain for FGD1 protein function. This study provides certain reference value for clinical diagnosis and genetic counseling of *FGD1*-related AAS patients.

### Supplementary Information

Below is the link to the electronic supplementary material.

Supplementary file1 (DOCX 657 KB)

Supplementary file2 (DOCX 656 KB)

## Data Availability

No datasets were generated or analyzed during the current study.

## Abbreviations

AAS: Aarskog–Scott syndrome
ACMG: The American College of Medical Genetics
ERK 1/2: Extracellular signal-regulated kinase 1/2
*FGD1*: FYVE, RhoGEF, and PH domain-containing 1
GEF: Guanine nucleotide exchange factor
GHD: Growth hormone deficiency
HGMD: The Human Gene Mutation Database
PEG-rhGH: PEGylated rhGH
rhGH: Recombinant human growth hormone
RUNX2: Runt-related transcription factor 2
